# Draft Genome Sequence of *Bacillus pumilus* Fairview, an Isolate Recovered from a Microbial Methanogenic Enrichment of Coal Seam Gas Formation Water from Queensland, Australia

**DOI:** 10.1128/genomeA.00279-14

**Published:** 2014-04-17

**Authors:** Cassandra J. Vockler, Paul Greenfield, Nai Tran-Dinh, David J. Midgley

**Affiliations:** a CSIRO Animal, Food and Health Sciences, North Ryde, NSW, Australia; b CSIRO Computational Informatics, North Ryde, NSW, Australia

## Abstract

Despite its global abundance, *Bacillus pumilus* is poorly studied. The Fairview strain was obtained from a methanogenic anaerobic coal digester. The draft genome sequence was 3.8 Mbp long and contained 3,890 protein-coding genes. Like the SAFR-032 strain, it includes *B. pumilus*-specific proteins that likely confer enhanced resistance to environmental stresses.

## GENOME ANNOUNCEMENT

*Bacillus pumilus* Fairview was isolated from a 45°C methanogenic anaerobic digester sample obtained from a coal seam gas formation water sample growing with a highly volatile bituminous Bowen Basin coal as the feedstock. The formation water was sourced from the Fairview coal seam gas field near Injune, Queensland, Australia (S25°51; E148°34) from a borehole intersecting a coal seam at approximately 600 m subsurface. On collection, the water was 45°C (pH 8.9) and had an electrical conductivity (EC) of 1,400 µS cm^–1^. The digester was incubated at 45°C for 8 weeks before attempts were made to culture microbes from the consortium. These efforts included dilution on a range of media, including peptone, yeast-extract, and glucose (PYG) (all 1 g liter^–1^) agar (1), followed by incubation to isolate facultative aerobes. One of the colonies isolated was the *B. pumilus* Fairview strain described. The pure culture was grown in liquid PYG, DNA was extracted, and Illumina (HiSeq, 100-bp paired-end [PE] library) sequencing was undertaken. Of the reads, 99.95% were assembled using Velvet 1/2/07 (*k* = 41) into the draft genome.

The draft genome of Fairview was 3,838,013 bp long and had coverage of ~200×. The genome was composed of 67 large contigs (>200 bp). Excluding the short contigs, mean and median contig lengths were 57,284 bp and 18,358 bp, respectively, with a maximum contig length of 568,093 bp. (The short contigs [<200 bp] are available at http://dx.doi.org/10.4225/08/531CF5598D431.) Genome annotation was undertaken with Integrated Microbial Genomes Expert Review (IMG ER) (2), which predicted 3,890 protein-coding genes. Based on coverage, we estimated that *B. pumilus* Fairview had five copies of the 16S gene. This 16S sequence has 99 to 100% identity with an array of *B. pumilus*, including the well-known SAFR-032 strain (3). Genomic comparisons of *B. pumilus* genomes available to date indicate that the Fairview strain is closely related to the CCMA-560 strain isolated from an oil-affected sediment (4) with which it shares ~89% of its genome. Further analyses of the genome using the SignalP (5) and dbCAN (6) pipelines revealed a small number of proteins which probably were extracellular β-glucosidase, pectin, or pectate lyase enzymes. Despite its presence in the digester, with coal as a sole source of carbon, no polyphenol oxidase or laccases were detected; however, a vanillyl-alcohol oxidase was identified and may be involved in some aromatic degradative processes.

It remains unclear whether the taxon was growing in the digestor or was present as a spore; genes for respiratory nitrate reduction were not detected. Furthermore, spores of *B. pumilus* have been shown to have extreme resistance to a range of stressors (7–9) and this may be conferred by a range of repair proteins unique to *B. pumilus* (3). Like SAFR-032, the Fairview strain possesses analogs of a *B. pumilus*-specific DNA photo-lyase enzyme (csirobg2_00392) that may confer resistance properties to this strain. Despite occurring in many environments, *B. pumilus* is relatively poorly studied compared to its relative *B. subtilis*. The ability of *B. pumilus* to survive in extreme environments, host unusual genes, and produce a range of metabolites warrants further examination.

### Nucleotide sequence accession numbers.

This whole-genome shotgun project has been deposited at DDBJ/EMBL/GenBank under the accession number JFBY00000000. The version described in this paper is the first version, JFBY01000000.
